# Single vs. multiple fraction non-inferiority trial of stereotactic ablative radiotherapy for the comprehensive treatment of oligo-metastases/progression: SIMPLIFY-SABR-COMET

**DOI:** 10.1186/s12885-024-11905-7

**Published:** 2024-02-03

**Authors:** Robert Olson, Hadassah Abraham, Curtis Leclerc, Alexander Benny, Sarah Baker, Quinn Matthews, Nick Chng, Alanah Bergman, Benjamin Mou, Emma M. Dunne, Devin Schellenberg, Will Jiang, Elisa Chan, Siavash Atrchian, Shilo Lefresne, Hannah Carolan, Boris Valev, Scott Tyldesley, Andrew Bang, Tanya Berrang, Haley Clark, Fred Hsu, Alexander V. Louie, Andrew Warner, David A. Palma, Doris Howell, Aisling Barry, Laura Dawson, Petra Grendarova, Debra Walker, Rishi Sinha, Jillian Tsai, Houda Bahig, Isabelle Thibault, Rashmi Koul, Sashendra Senthi, Iain Phillips, Derek Grose, Paul Kelly, John Armstrong, Ronan McDermott, Candice Johnstone, Srini Vasan, Noel Aherne, Stephen Harrow, Mitchell Liu

**Affiliations:** 1 University of British Columbia, Vancouver, Canada; 2 University of Northern British Columbia, Prince George, Canada; 3 BC Cancer – Prince George, 1215 Lethbridge Street, Prince George, BC V2M7A9 Canada; 4 BC Cancer – Surrey, Surrey, British Columbia Canada; 5 BC Cancer – Vancouver, Vancouver, British Columbia Canada; 6 BC Cancer – Kelowna, Kelowna, British Columbia Canada; 7 BC Cancer- Victoria, Victoria, British Columbia Canada; 8 BC Cancer- Abbotsford, Abbotsford, British Columbia Canada; 9 Department of Radiation Oncology, Odette Cancer Centre, Sunnybrook Health Sciences Centre, Toronto, Ontario Canada; 10 Department of Oncology, London Health Sciences Centre, London, Ontario Canada; 11 Princess Margaret Cancer Centre, Toronto, Ontario Canada; 12 Cork University Hospital, Cork, Ireland; 13 Patient partner, BC Cancer-Prince George, Prince George, BC Canada; 14 Tom Baker Cancer Centre, Calgary, Alberta Canada; 15 Centre Hospitalier de l’Université de Montréal (CHUM), Montréal, Québec Canada; 16 Centre Hospitalier Universitaire de Québec (CHUQ), Québec, Canada; 17 Cancer Care Manitoba, Winnipeg, Manitoba Canada; 18 Alfred Health Radiation Oncology, Melbourne, Australia; 19 Western General Hospital/Edinburgh Cancer Centre, Edinburgh, Scotland; 20 Beatson West of Scotland Cancer Centre, Glasgow, Scotland; 21 Bon Secours Radiotherapy Cork (In Partnership with UPMC Hillman Cancer Centre), Cork, Ireland; 22 St. Luke’s Radiation Oncology Network, Dublin, Ireland; 23 University Hospital Galway, Galway, Ireland; 24 Medical College of Wisconsin, Milwaukee, Wisconsin United States of America; 25 Precision Cancer Center, Ashland, Kentucky United States of America; 26 Riverina Cancer Care Centre, Wagga Wagga, New South Wales Australia; 27 Department of Radiation Oncology, BC Cancer – Centre for the North, 1215 Lethbridge Street, Prince George, British Columbia V2M 7E9 Canada

**Keywords:** Stereotactic ablative radiotherapy, Oligometastases, Quality of Life, Survival, Cancer, Single Fraction

## Abstract

**Background:**

Radiotherapy delivery regimens can vary between a single fraction (SF) and multiple fractions (MF) given daily for up to several weeks depending on the location of the cancer or metastases. With limited evidence comparing fractionation regimens for oligometastases, there is support to explore toxicity levels to nearby organs at risk as a primary outcome while using SF and MF stereotactic ablative radiotherapy (SABR) as well as explore differences in patient-reported quality of life and experience.

**Methods:**

This study will randomize 598 patients in a 1:1 ratio between the standard arm (MF SABR) and the experimental arm (SF SABR). This trial is designed as two randomized controlled trials within one patient population for resource efficiency. The primary objective of the first randomization is to determine if SF SABR is non-inferior to MF SABR, with respect to healthcare provider (HCP)-reported grade 3-5 adverse events (AEs) that are related to SABR. Primary endpoint is toxicity while secondary endpoints include lesional control rate (LCR), and progression-free survival (PFS). The second randomization (BC Cancer sites only) will allocate participants to either complete quality of life (QoL) questionnaires only; or QoL questionnaires and a symptom-specific survey with symptom-guided HCP intervention. The primary objective of the second randomization is to determine if radiation-related symptom questionnaire-guided HCP intervention results in improved reported QoL as measured by the EuroQoL-5-dimensions-5levels (EQ-5D-5L) instrument. The primary endpoint is patient-reported QoL and secondary endpoints include: persistence/resolution of symptom reporting, QoL, intervention cost effectiveness, resource utilization, and overall survival.

**Discussion:**

This study will compare SF and MF SABR in the treatment of oligometastases and oligoprogression to determine if there is non-inferior toxicity for SF SABR in selected participants with 1-5 oligometastatic lesions. This study will also compare patient-reported QoL between participants who receive radiation-related symptom-guided HCP intervention and those who complete questionnaires alone.

**Trial registration:**

Clinicaltrials.gov identifier: NCT05784428. Date of Registration: 23 March 2023.

**Supplementary Information:**

The online version contains supplementary material available at 10.1186/s12885-024-11905-7.

## Background

The oligometastatic state refers to a stage of disease where a cancer has spread beyond the site of the primary tumour, but is not yet widely metastatic [1]. In patients with a limited oligometastatic burden, emerging evidence suggests that treatment of all sites of disease with ablative therapies such as surgery or stereotactic ablative radiotherapy (SABR) can improve patient outcomes, including overall survival (OS) and progression-free survival (PFS) [2, 3]. The oligoprogressive state is similar to the oligometastatic state; however, there are some key distinctions. Oligoprogression is the progression of limited metastases following an initial response to systemic treatment [2]. In contrast to the oligometastatic state, the oligoprogressive state can have any number of metastases as long as these were, at one point, controlled [3]. The oligoprogressive state is being seen more often in clinical encounters [2], potentially due to increased utilization of targeted therapies and subsequent acquired resistance of a subpopulation of tumour cells [2, 4]. Recent research has shown that stereotactic ablative strategies might be appropriate for the treatment of the oligoprogressive state although less evidence exists than for the oligometastatic state [5].

Historically, treatment for patients with both oligometastatic and oligoprogressive cancers have been predominantly based on systemic therapies (chemotherapy, hormonal, targeted and immunotherapies) with the intent to delay progression and extend life. However, emerging evidence, suggests that treatment of all oligometastatic sites with ablative therapies such as surgery or SABR are associated with favorable outcomes [6–8]. SABR is a modern technique that delivers high doses of radiotherapy (RT) to tumour targets using highly conformal techniques. Modern SABR techniques effectively limit dose to nearby organs while escalating the tumour dose and achieving excellent rates of local control. However, as there is a potential for higher toxicity, SABR requires extensive planning and time on the treatment unit to deliver each fraction safely, compared to conventional RT, thus resulting in higher costs. Based upon existing randomized evidence supporting SABR for oligometastatic cancer and emerging evidence for benefit also in the oligoprogressive state, we should explore optimizing SABR treatment regimens in order to maintain favorable toxicity profiles and oncologic outcomes while potentially improving cost-efficacy and patient convenience.

RT delivery regimens can vary between a single fraction (SF) and multiple fractions (MF) given daily for up to several weeks depending on the location of metastases, while SABR is generally limited to eight or fewer fractions. MF RT is administered to reduce the radiation load given at a single event, which may reduce late toxicities. However, SF RT may be more cost effective and reduces patient treatment time. While SF SABR is a safe and effective treatment for brain metastases, the toxicity profile for other metastatic sites and in the setting of oligometastases is not well established. One study comparing SF with MF SABR in treatment of 1-3 pulmonary oligometastases showed no statistically significant difference in toxicities or OS between the groups, however, this was a retrospective study and limited to a small sample size [9]. The recent randomized phase 2 SAFRON II trial reported no difference between SF and MF SABR for lung oligometastases in the primary endpoint of severe toxicity, and also similar local control, survival and qualify-of-life (QoL) between treatment arms [10, 11]. Another recently completed phase III randomized controlled trial (RCT) comparing local control of oligometastases between SF and MF SABR found significantly lower cumulative incidence of local recurrence in the SF arm [12]. However, the biologically equivalent dose for the MF arm was significantly lower than for the SF arm, thereby limiting the study’s interpretation and generalizability. We have found no studies that have investigated SF vs. MF for oligoprogression. Consensus among radiation oncologists and our patient partners was to perform a study with toxicity as the primary outcome given concerns that SF SABR may have a higher rate of toxicity to nearby organs at risk. It is critical that a properly powered phase III trial is conducted comparing toxicity, efficacy, and cost-effectiveness between SF and MF SABR with similar biologically effective doses, a larger sample size, and inclusive of all tumour sites to expand our knowledge of SABR in both oligometastatic and oligoprogressive settings and to guide evidence-based prescription of SF SABR.

In a study by Basch et al., changes in QoL in patients receiving outpatient chemotherapy for advanced solid tumours were compared [13]. Patients were randomly assigned to complete symptom monitoring electronically, or received standard of care, and results in this systemic therapy population showed a greater improvement in QoL and OS from baseline to 6 months in the symptom monitoring arm. Our study will implement similar methodology in the radiotherapy setting. We will examine differences in patient-reported QoL in the second randomization, comparing change in EQ-5D-5L scores between those who complete questionnaires alone and those who complete questionnaires, a symptom-specific survey and receive HCP-guided intervention.

## Methods

The objective of this trial is to assess if there is non-inferiority in terms of HCP-reported grade 3 or higher toxicities, lesional control rate (LCR), PFS, cost-effectiveness, and OS in patients with 1-5 oligometastatic or oligoprogressive lesions receiving SF SABR compared to patients receiving MF SABR. The trial will also assess if radiation-related symptom questionnaire-guided HCP intervention provides improvement in patient-reported QoL, compared to no HCP intervention in a subset of sites. The methods to be employed in this trial follow similar methodology used in our current SABR-COMET-3 trial [14].

### 1st randomization

#### Primary endpoint

##### Toxicity

- ◦ Defined as grade 3-5 adverse events (Common Terminology Criteria for Adverse Events [CTCAE] version 5.0) that are possibly, probably, or definitely related to SABR.

#### Secondary endpoints

##### LCR

- ◦ Assessed using the Tumour Lesion Measurement form. Rate is determined based on lesion size post-SABR using Response Evaluation Criteria in Solid Tumours (RECIST) version 1.1.

##### OS (exploratory)

- ◦ Time from randomization to death from any cause, or last follow-up, whichever occurs first.

##### PFS

- ◦ Time from randomization to disease progression at any site, death, or last follow-up, whichever occurs first.

##### QoL

- ◦ Assessed via Functional Assessment of Cancer Therapy: General (FACT-G)

##### QoL

- ◦ Assessed via EQ-5D-5L questionnaire.

##### Resource utilization

- ◦ Assessed via Patient- and Provider-Reported number of hospital admissions, emergency room (ER) visits, systemic or RT.

### 2nd Randomization (BC Cancer sites only)

#### Primary endpoint

##### Differences in patient reported QoL

- As measured by the EQ-5D-5L based on presence or absence of HCP intervention (guided by symptoms reported in the generic radiation-related symptom questionnaire).

#### Secondary endpoints

##### Patient hospitalization and emergency department visit rates

- ◦ Captured in self-reported forms and as reported by the HCP.

##### Cost-effectiveness of Patient Reported Outcome (PRO)-guided intervention

- ◦ Assessed via Patient- and Provider-Reported resource utilization forms

##### OS (exploratory)

- ◦ Time from randomization to death from any cause, or last follow-up, whichever occurs first.

## Study design

**Fig. 1:**
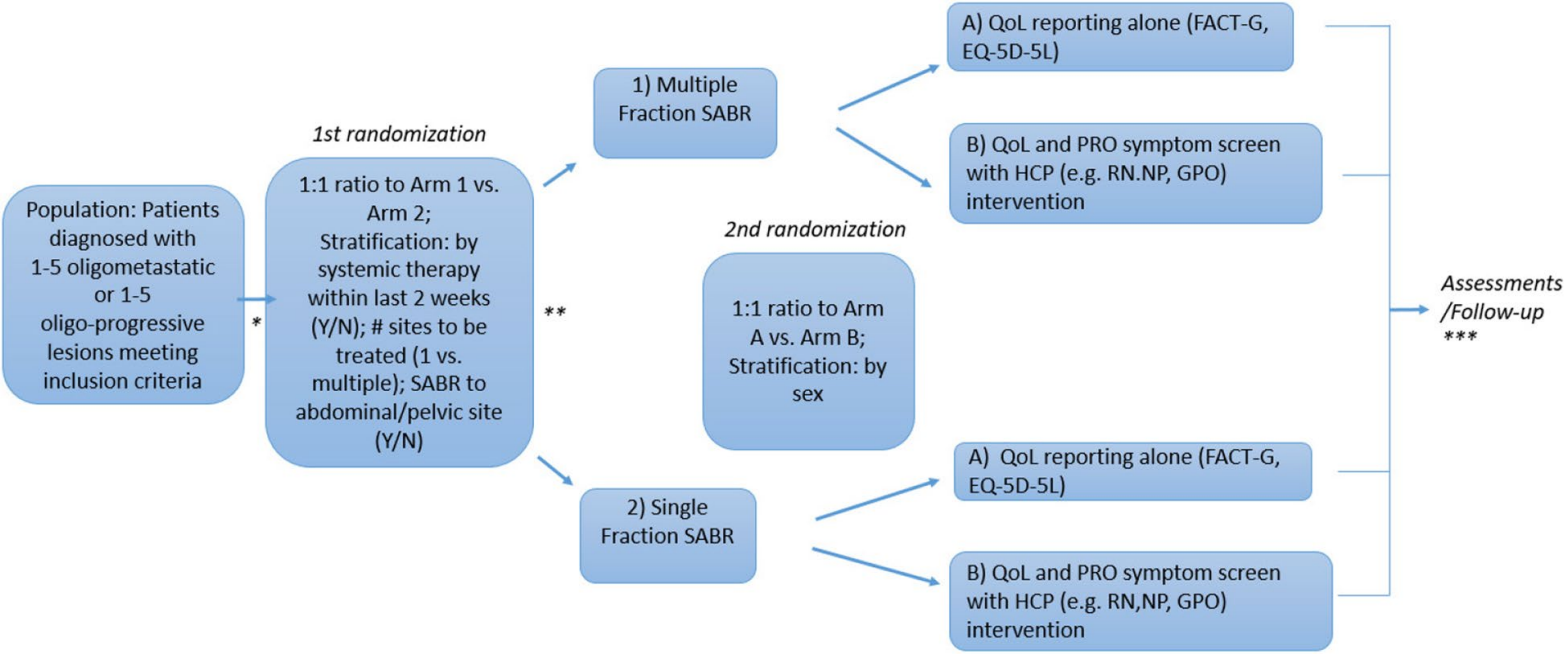
Study schema

This is a phase III, investigator-led, open-label, multi-institutional, RCT using a 2x2 factorial design with two randomizations and two primary outcomes. Participating institutions will be tertiary, academic hospitals or RT treatment institutions in Canada, the United Kingdom, United States, and Australia (updated country list available on ClinicalTrials.gov entry NCT05784428). The first and primary randomization is by dose and fractionation, while the second randomization is by HCP-led intervention based on PRO symptom screen, as displayed in schema Fig. 1.

## Inclusion criteria

- ◦ Total number of current metastases of 1-5 (either oligometastatic or oligoprogressive lesions)
- ◦ Age 18 years or older
- ◦ Able to provide informed consent
- ◦ Able to complete electronic entry (mandatory for BC Cancer sites) or paper entry of patient-reported outcomes independently or with assistance from a caregiver/family/friend/research staff using electronic methods after providing consent for use of email
- ◦ Eastern Cooperative Oncology Group (ECOG) performance status 0 – 2
- ◦ Life expectancy > 6 months
- ◦ Histologically confirmed malignancy with metastatic disease detected on imaging
  - Biopsy of metastasis is preferred, but not required
- ◦ Controlled primary tumour
  - defined as: at least 3 months since original tumour treated definitively, with no progression at primary site (can be considered controlled if no evidence of primary tumour on imaging [e.g. primary unknown])
- ◦ A history and physical examination including ECOG performance status performed within 6 weeks prior to trial enrollment
- ◦ Patient has had a computed tomography (CT) chest, abdomen and pelvis or positron emission tomography (PET-CT) within 8 weeks prior to enrollment, and within 12 weeks prior to treatment. CT neck as clinically indicated
- ◦ Patient has had a nuclear bone scan (if no PET-CT) within 8 weeks prior to enrollment, and within 12 weeks prior to treatment
- ◦ If solitary lung nodule for which biopsy is unsuccessful or not possible, patient has had an fluorodeoxyglucose (FDG) PET scan or CT (chest, abdomen, pelvis) and bone scan within 8 weeks prior to enrollment, and within 12 weeks prior to treatment. CT Neck as clinically indicated
- ◦ If colorectal primary with rising carcinoembryonic antigen (CEA), but equivocal imaging, patient has had an FDG PET scan within 8 weeks prior to enrollment, and within 12 weeks prior to treatment
- ◦ Patient has had CT or magnetic resonance imaging (MRI) of brain if primary has a propensity for central nervous system (CNS) metastasis within 8 weeks prior to enrollment, and within 12 weeks prior to treatment
- ◦ For patients with known spine metastases, patient has had MRI spine imaging within 8 weeks prior to enrollment, and within 12 weeks prior to treatment.
- ◦ Patient is judged able to:
  - Maintain a stable position during therapy.
  - Tolerate immobilization device(s) that may be required to deliver SABR safely
- ◦ Negative pregnancy test for People of Child-Bearing Potential (POCBP) ***within 4 weeks of RT start date***

## Exclusion criteria

- ◦ Uncontrolled concurrent malignant cancer
- ◦ Lesion in femoral bone requiring surgical fixation
- ◦ Serious medical comorbidities precluding RT. These include interstitial lung disease in patients requiring thoracic radiation, Crohn’s disease in patients where the gastrointestinal (GI) tract will receive RT, and connective tissue disorders such as lupus or scleroderma.
- ◦ Substantial overlap with a previously treated radiation volume. Prior RT in general is allowed, as long as the composite plan meets dose constraints herein. For patients treated with conventional radiation previously, similar biologically effective dose calculations should be used to equate previous doses to the tolerance doses listed below. All such cases should be discussed with the local Principal Investigators (PIs) and Sponsor Investigator.
- ◦ Current malignant pleural effusion
- ◦ Liver metastases + planning tumour volume (PTV) located within the “Biliary no fly zone” defined for this trial as the central biliary tract (CBT) (common biliary tract, cystic duct and 1 cm of distal branches) + 1 cm (i.e. CBT + 1 cm = biliary no fly zone)
- ◦ Inability to treat all sites of oligometastatic or oligoprogressive disease
- ◦ Lesions greater than 5 cm outside the brain, except:
  - ◦ Bone metastases over 5 cm may be included, if in the opinion of the local PI it can be treated safely (e.g. rib, scapula, pelvis)
  - ◦ Any brain metastasis > 3.5 cm in size or a total volume of brain metastases greater than 30 cc
- ◦ Clinical or radiologic evidence of spinal cord compression. Patients can be eligible if surgical resection has been performed.
- ◦ Patients with spine instability as judged by a Spinal Instability Neoplastic Score (SINS) of > 12 [15]
- ◦ Dominant brain metastasis requiring surgical decompression
- ◦ Surgical resection of all metastases (i.e. no lesion available to be treated with SABR)
- ◦ Pregnant or breast feeding

## Pre-treatment evaluation

- ◦ History and Physical Examination within 6 weeks of study accrual:
  - ◦ Including prior cancer therapies and cancer-specific concomitant medications (for example, systemic therapy such as immunotherapy, hormone therapy and/or chemotherapy drugs and regular/supporting medications such as anti-emetics) [14].
- ◦ Re-staging within 8 weeks prior to randomization, and within 12 weeks of treatment:
  - ◦ Brain: CT or MRI for tumour sites with propensity for brain metastasis. All patients with brain metastases at enrollment or previously require an MRI.
  - ◦ Body: 18-FDG PET/CT imaging is strongly recommended, except for tumours where FDG uptake is not expected (e.g. prostate, renal cell carcinoma). Prostate-specific membrane antigen (PSMA)-PET or choline-PET is recommended for prostate cancer. In situations where a PET scan is unavailable, or for tumours with limited radiotracer uptake, CT chest/abdomen/pelvis with bone scan required. CT neck as clinically indicated.
  - ◦ Spine: MRI is required for patients with vertebral or paraspinal metastases, though the MRI can be limited to the involved segment, including at least the involved vertebral body(ies) plus at least one vertebral body above and below, where applicable [14].
- ◦ Pregnancy test for women of child-bearing potential within four weeks of RT start date [14].

### Defining the number of metastases

Patients are eligible if there are 1-5 current oligometastatic lesions present. For oligoprogression, patients can have more than 5 lesions, but only 5 or fewer can be growing, and considered oligoprogressive lesions. Each discrete lesion is counted separately. For patients with lymph node metastases, each node is counted as one site of metastasis. All known metastatic lesions must be targetable on planning CT. For patients where the lesion is only detectable on MRI, fusion of the MRI with the planning CT is required [14].

Patients with prior metastases that have been treated with ablative therapies (e.g. SABR, surgery, radiofrequency ablation) are eligible, as long as those metastases are controlled on imaging. Brain metastases have to have been treated, or will be treated, with ablative technique (surgery, stereotactic radiosurgery [SRS], or stereotactic radiotherapy [SRT]).

When patients have small indeterminate nodules (e.g. a 2 mm lung nodule) it can be difficult to determine whether these are benign or whether they represent metastasis. Any such lesion that is ‘new’ is automatically considered a metastasis unless there are > 2 months of documented stability without systemic therapy [14].

### Brain metastases at presentation

If a patient presents with 1-5 brain metastases and ablation of those metastases (with surgery or radiation) is judged to be clinically required regardless of the treatment of extracranial metastases, ablative treatment is permitted. Those treated metastases count within the total number of five lesions. The patient would then be randomized to treatment of the extracranial disease. For example, a patient with a solitary brain metastasis and two lung metastases could receive an ablative technique to the brain (e.g. surgery, SRS, or fractionated stereotactic radiotherapy [FSRT]), and then be randomized to SF SABR vs. MF SABR for the two lung metastases.

### Patients already receiving systemic therapy

If a patient is already receiving systemic therapy, they are still eligible for enrolment. For example, if a patient with four metastases has been on systemic therapy for a year and is planning to continue, they can still be randomized, and will receive SABR (either SF or MF, dependent upon randomization) between cycles, and may require a short treatment break.

## Interventions

### Standard arm – MF SABR (ARM 1)

Patients in the standard arm will receive MF SABR with doses dependent upon the tumour site, as shown in Table 1.

**Table 1:** Dose and fractionations in standard of care arm (multi-fraction) by site with [secondary options in square brackets]

**Location**	**Dose (Gy)**	**Fractions**	**Dose per fraction (Gy)**	**Tumour BED (Gy**_**10**_**)**	**Frequency**
**Lung** Greater than 2 cm from mediastinum or brachial plexus or if mandatory OAR constraints are met	48 [54]	4 [3]	12 [18]	105.6 [151.2]	Daily or every second day
Within 2 cm of mediastinum or brachial plexus	60 [50]	8 [5]	7.5 [10]	105 [100]	Daily
**Bone** Any bone except spine	35	5	7	59.5	Daily
**Liver**	54 [50]	3 [5]	18 [10]	151.2 [112.3]	Daily or every second day
**Spine**	24 [35]	2 [5]	12 [7]	52.8 [59.5]	Daily
**Adrenal**	40 [35]	5	8 [7]	72 [59.5]	Daily
**Lymph node/soft tissue**	40 [35]	5	8 [7]	72 [59.5]	Daily
**Brain**	Dose per institutional policy				

### Experimental arm – SF SABR (ARM 2)

Patients in the experimental arm will receive SF SABR with doses dependent upon tumour site, as shown in Table 2.

**Table 2:** Dose and fractionation in experimental arm (Single Fraction), with [secondary options in square brackets]

**Location**	**Dose (Gy)**	**Fractions**	**Tumour BED (Gy**_**10**_**)**
**Lung** Greater than 2 cm from mediastinum or brachial plexus or if mandatory OAR constraints are met	30 [34]	1	120 [149.6]
Within 2 cm of mediastinum or brachial plexus	20	1	60
**Bone** Any bone	20	1	60
**Liver**	30 [34]	1	120 [149.6]
**Spine**	20	1	60
**Adrenal**	20	1	60
**Lymph node/ soft tissue**	20	1	60
**Brain**	Dose per institutional policy	1	

### Standard arm – QoL questionnaires only (ARM A)

0-3 days prior to each scheduled patient visit on trial, patients will be prompted to complete the EQ-5D-5L and FACT-G questionnaires [16, 17]. Questionnaire data will be saved in the study database.

### Experimental arm – QoL questionnaires and symptom-specific intervention (ARM B)

0-3 days prior to each scheduled study visit on trial, patients will be automatically prompted to complete the EQ-5D-5L, FACT-G as well as a PRO symptom screen utilizing a generic radiation-related symptom questionnaire. In addition, patients may report symptoms *ad hoc,* with a response by the HCP within three working days. Depending on symptoms entered, patients may receive HCP-guided intervention such as symptom management advice or referral to family physician.

### Immobilization

Treatments will be set up using reproducible positioning and verified using an on-line imaging protocol for all patients in this study. Immobilization may include a custom immobilization device, such as thermoplastic shell or vacuum bag, as per individual institutional practice when delivering SABR. Some institutions do not use immobilization devices and have demonstrated high degrees of accuracy; this is acceptable in this study. Immobilization techniques and devices must not differ between the trial arms.

### Simulation imaging/localization/registration

All patients will undergo planning CT simulation. 4-dimensional (4D) CT will be used for tumours in the lungs, liver, or adrenals, and should be considered for mobile rib lesions. Axial CT images will be obtained throughout the region of interest. The maximum CT slice thickness should be 2.5 mm. The simulation approach for a given site must not differ between trial arms.

### 4D-CT procedures

For patients undergoing 4D-CT for motion management, medical physics will review the 4D-CT images:

i) If the quality of the 4D-CT phase bin images is not sufficient, or fails to reconstruct, then untagged average CT and Maximum Intensity Projection (MIP) (± Minimum Intensity Projection (MinIP)) images should be provided for the treatment planning system. Planning will be performed on average CT or 3D helical CT per physics discretion. Physics will assist in assessing the total target motion based on the available scans.
ii) Motion assessments in all 3 directions are performed:
  1. If the motion is less than or equal to 10 mm, then treatment planning for the motion encompassing method (internal tumour volume [ITV] method) may be performed. Generally, the average CT is used for planning, but planning scan may be a 3D helical per institutional policy.
    The 50%-60% phase (end expiration) and the 0%-10% phase (end inspiration) should be fused to the planning scan to help define the ITV.
  2. If the motion is greater than 10 mm in any one direction, then respiratory-gated or dynamic tumour tracking RT can be considered. Planning CT will be per departmental protocol for the technique selected (generally breath hold exhale scan or 4D-CT 50% phase bin (exhale)).
  3. If the motion is greater than 10 mm in any one direction, and respiratory-gated or dynamic tumour tracking RT is not available, ITV method can be considered. Assessing nearby organ at risk (OAR) motion is required and planning at risk volumes (PRVs) applied as appropriate.

### Volume definitions (arms 1 & 2)

For all lesions, the gross tumour volume (GTV) will be defined as the visible tumour on CT and/or MRI imaging ± PET. No additional margin is required for microscopic spread of disease (i.e. clinical target volume [CTV] = GTV) for non-bony lesions). For bone lesions, CTV of 5-10 mm is advised, but 3-5 mm will be allowed upon treating investigator’s discretion. CTV delineation for non-spine bone metastases will be based on consensus recommendations from international experts as published elsewhere [18]. For vertebral lesions, an anatomic approach will be taken as per the International Spinal consortium guidelines [19–21].

An anatomic approach is taken to the CTV based on where the diseases within the spinal segment are located. The rules for CTV are as follows:

1. If the vertebral body is involved with GTV then the entire vertebral body is taken as CTV.
2. If the ipsilateral pedicle and/or transverse process has GTV then the entire ipsilateral posterior segment (pedicle, lamina and transverse process) ± the spinous process is taken into the CTV. The inclusion of the spinous process is per the discretion of the radiation oncologist.
3. If the ipsilateral pedicle, lamina, and/or transverse process has GTV, then the entire ipsilateral posterior segment (pedicle, lamina, and transverse process) plus the spinous process is taken into the CTV
4. If there is bilateral involvement of the pedicles and/or transverse processes with GTV, then the posterior segment anatomy ± the spinous process is taken into the CTV. The inclusion of the spinous process is per the discretion of the radiation oncologist.
5. If there is bilateral involvement of the pedicles and laminae, and/or transverse processes with GTV, then the entire posterior segment anatomy is taken into the CTV, including the spinous process.
6. If the spinous process is involved with GTV alone then the bilateral lamina ± pedicles are to be taken into the CTV.

The International Spinal Consortium Guideline is a reference for CTV delineation and can be adhered to as they have described [19, 20].

PTV margins of up to 5 mm will be added depending on site of disease, immobilization, and institutional set-up accuracy: 1-3 mm margins may be used for spinal stereotactic treatments, 0-2 mm for brain tumours, and 3-5 mm (dependent on institutional policies) for other sites. PTV margins for a given site must not differ between trial arms.

### OAR doses

OAR doses are listed in the section “Figures, Tables and Additional Files”. OAR doses may not be exceeded except in the case of chest wall/rib(s) involvement. In cases where the PTV coverage cannot be achieved without exceeding OAR doses, the PTV coverage is to be compromised. All serial organized OARs within 5 cm of the PTV must be contoured (partial organ contours allowed); for parallel organized organs (liver, lung, etc.) within 5 cm of PTV, the whole organs need to be contoured. This should be tested for each PTV by creating a 5 cm expansion to examine which OARs lie within that expansion. This applies to volumetric modulated arc therapy deliveries only. For static, multi-beam field deliveries, care should be made to ensure all organs that intersect the beam meet dose constraints (i.e. no unanticipated hot areas along beam path, such as hot-spots in skin).

Organs that are under the influence of motion (i.e. respiratory) must be considered for the addition of a PRV, particularly for the motion encompassing (ITV) treatment delivery method. PRV size will be determined by radiation oncologist with input from physics. The approach to determining PRV margins must not differ between trial arms.

If OAR motions are affected by respiration (e.g. in treating thoracic or upper abdominal targets), the OARs should be contoured with the help of 4D-CT (and use of MIP for soft tissue OARs and MinIP for airways OARs).

Use of an OAR PRV (~2-3mm) should also be strongly considered if the risk of high grade toxicities is perceived to be high, for example when treating central lung lesions (esophagus, proximal trachea/proximal bronchial trees, pulmonary arteries), left lower lobe or left adrenal lesions (stomach if close to the target), or abdominal/pelvic lesions (GI tract OARs including esophagus, stomach, small/large bowels, central biliary tracts, etc).

For vertebral tumours, a spinal cord PRV is strongly recommended. For other sites, either a spinal cord PRV or a spinal canal contour are acceptable.

### Treatment planning

Treatment can be delivered using static beams (either 3D-conformal radiotherapy or intensity-modulated) or rotational therapy (volumetric modulated arc therapy, 3D conformal arcs or tomotherapy). Systematic differences in treatment techniques between trial arms for a particular treatment site are not permitted.

OAR dose constraints may not be exceeded (except chest wall/ribs). If a dose constraint cannot be achieved due to overlap of the target with an OAR, the fractionation (for patients in Arm 1) can be increased, or the target coverage compromised in order to meet the constraint. In cases where the target coverage or dose must be reduced, the priority for dose coverage is the GTV (e.g. attempt to cover as much of the GTV as possible with the prescription dose). All such cases of dose reduction or target coverage compromise must be approved by the local PI prior to treatment.

For patients treated with radiation previously using a different fractionation, similar biologically effective dose calculations should be used to equate previous doses to the tolerance doses listed in Appendix 1 and 2 (with the exception of mean lung dose tolerances, which may be excluded in the context of prior conventionally fractionated radiation doses summed with SABR treatment doses). Equivalent Dose in 2 fractions (EQD2) is the standard radiobiology conversion for overlapping treatment plans contributing to an accumulated dose (sum). An α/β = 2 for cord/brainstem/nerve structures and α/β =3 for all other normal tissues is the current standard.

“Hot Spot” is defined as the dose to 0.035cc volume.

For all targets, doses should be prescribed to either using: 1) the prescription isodose method or 2) the dose-volume method. For prescription isodose method, dose is prescribed to the 60–90% isodose line surrounding the PTV, and all hotspots should fall within the GTV or ITV. For the dose-volume method, ≥ 95% of the PTV should be covered by 100% of the prescription dose, and at least 99% of the PTV should be covered by 90% of the prescription dose. The hot spot should be less than or equal to 150% of the prescription dose (at treating physician’s discretion, up to 167% is acceptable) and should fall within the GTV or ITV.

For spine metastases, it is recommended to keep the hotspots of CTV not including “GTV + 2 mm” to < 110%, to reduce the risk of vertebral compression fracture (VCF).

It is recommended to use the PRV and strict Image-guided radiation therapy (IGRT) credentialing to have strong confidence with small uncertainty that the maximum doses are not much higher than the prescribed volume.

Dose calculation algorithms should be “Type B” (convolution/superposition) or “Type C” (radiation transport equations or Monte Carlo). Doses must be corrected for inhomogeneous tissues (i.e. lung, bone). Dose grid resolution should be ≤ 2.5 mm. For spine and brain a dose grid of ≤ 1.25 mm is recommended. Dose calculation algorithms may differ between treatment sites (for example Type C may be used in lung but not in the pelvis) but are not permitted to differ between trial arms for the same site.

The number of isocentres is at the discretion of the treating physician, physicists, and dosimetrists. Generally, metastases are treated with separate isocentres if they are well-separated. Single isocentre, multiple PTV plans are allowed if approved by physics.

Image guidance is required for SABR delivery. Cone Beam CT (CBCT) volumetric imaging is recommended, but orthogonal KV imaging is allowed per discretion of medical physicist, physician and treating radiation therapists.

Image guidance (kV vs. CBCT) must not systematically differ between trial arms. However, for SF SABR (Arm 2), if intrafraction motion assessment and management (e.g., surface-guided RT, tumour tracking, etc.) is not available, the addition of repeat image guidance and re-positioning at mid-treatment is strongly recommended to correct for the increased risk of intrafraction motion.

The scheduling and sequence of treating each metastasis is at the discretion of individual physicians, but in general should begin with the brain, due to risks associated with progression. Radiation schedule will depend on sites of tumour being treated, but generally daily or every other day for 1-3 weeks.

### Quality Assurance (QA) (arms 1 & 2)

In order to ensure patient safety and effective treatment delivery, a robust quality assurance protocol is incorporated. The following requirements must be completed for each patient:

- Prior to treatment, each patient must be discussed at QA rounds or be peer reviewed by a radiation oncologist with SABR expertise.
- All RT plans must meet target dose levels for OARs (except chestwall/ribs) (section “Figures, Tables, and Additional Files”). Prior to plan approval, the dose to each OAR must be reviewed by the physicist or treating physician.
- All treatment plans must undergo physics QA review per institutional practice. The QA review must include the treatment beam monitor unit verification.
- All SABR treatments must be delivered on a RT linac commissioned for SABR use and complies with a QA schedule as recommended by current international published guidelines [22].

### Systemic therapy

Patients treated with prior systemic therapy are eligible for this study, however, no chemotherapy agents (cytotoxic, or molecularly targeted agents) are allowed within the period of time commencing one week prior to radiation lasting until one week after the last fraction. Hormonal therapy is allowed. Use of chemotherapy schemes containing potent enhancers of radiation damage (e.g. gemcitabine, adriamycin/doxorubicin, bevacizumab) should not be used within one week prior to SABR and are discouraged within the first four weeks after radiation (i.e. omit one cycle).

### Further RT for progressive disease at new metastatic sites

Patients in Arm 1 who develop new, untreated metastatic deposits should be considered for MF SABR at those sites, if such deposits can be treated safely with MF SABR, and if the treating institution offers MF SABR for that body site. If MF SABR is not possible, then SF SABR or palliative RT can be delivered as clinically appropriate.

Patients in Arm 2 who develop new, untreated metastatic deposits should be considered for SF SABR at those sites, if such deposits can be treated safely with SF SABR, and if the treating institution offers SF SABR for that body site. If SF SABR is not possible, then MF SABR or palliative RT can be delivered as clinically appropriate.

### QA for institutions joining study

Prior to opening the study, each participating research institution will be required to send to the Sponsor Investigator a mock treatment plan for the anatomic sites that will be treated (e.g. lung, brain, liver, adrenal), to ensure that the treatment plans are designed in compliance with the protocol. The local PIs will provide pertinent CT datasets. Each participating research institution can choose which tumour sites will be treated at their individual institution (i.e. some institutions may only choose to treat a subset of the eligible metastatic sites). Sites that have prior accreditation for SABR through a clinical trial (e.g. SABR-COMET, or organ-specific SABR trials) are exempt from this requirement for the organ sites that have been accredited in those trials.

### QA for 2^nd^ randomization

Clinical trial staff will provide a 5-20 minute training session to participants randomized to Arm B on how to use a patient outcomes management tool for self-reporting of symptoms at time of enrollment. Electronic entry platform will be configured to send an email alert to a HCP when a patient-reported symptom meets notification criteria.

## Patient discontinuation / withdrawal

Patients may discontinue participation in the study at any time. The clinical and laboratory evaluations that would have been performed at the end of the study should be obtained. If a subject is removed because of an adverse event, they should remain under medical observation as long as deemed appropriate by the treating physician. Patients withdrawn or discontinued can be replaced at the discretion of the Study Principal Investigator [14].

## Follow-up evaluation and assessment of efficacy

There is no planned follow-up at the end of the study. Additional care that will be provided to subjects, after they complete or discontinue the study, will involve the standard of care treatment for what is normally expected for their condition.

## Statistical considerations

### Randomization

#### 1^st^ randomization

The study will employ a 1:1 randomization between Arm 1 and Arm 2, with further stratification between 1) systemic therapy within last two weeks (yes vs. no), 2) number of sites to be treated with SABR (one vs. multiple) and 3) SABR to abdominal sites (yes vs. no).

Permuted block randomization will be used to reduce selection bias, promote allocation concealment, and improve balance across groups over the trial period. Patients will be randomized based on a permuted block design using a block size based on a multiple of 3 (with block size known only to statistician until analysis is completed [23]. The Coordinating Centre and trial statistician will be responsible for configuring the module within REDCap, the electronic data capture (EDC) system that will be used to randomize participants.

#### 2^nd^ randomization

The 2^nd^ randomization of the study will also employ a 1:1 randomization between Arm A and Arm B, with stratification by sex (female vs. male vs. other).

### Sample size calculation

#### 1^st^ randomization

The sample size is based on toxicity of SABR (grade 3-5 AEs possibly, probably, or definitely related to SABR collected by HCP). The previously published SABR-5 protocol pre-defined a 10% rate of grade ≥ 4 AEs as acceptable, and we will conservatively set a 10% grade ≥ 3 AE rate as acceptable for this study [8]. The standard and intervention arms will be accrued 1:1. Sample sizes of 299 in Arm 1 and 299 in Arm 2 are required to achieve 80% power to detect a non-inferiority margin difference of 5%, with the reference group grade ≥ 3 AEs set at 5% based on the results of SABR-5. Based on our clinical judgement, the treatment group is assumed to be 10% under the null hypothesis of inferiority and coincides with our pre-defined < 10% grade ≥ 3 AE rate. The power was computed for the case when the actual treatment group proportion is 5%. This was based on a one-sided z test (unpooled) and alpha of 0.025.

In addition, given the importance of efficacy as a secondary outcome, we wanted to ensure that this sample size would have sufficient power to rule out large effect size differences in LCR. Based on our SABR-5 trial data, the estimated LCR at 1 year is 90%. With 584 patients, assumed to be accrued over five years with an additional five years of follow-up, then we would achieve > 80% difference to rule out a non-inferiority hazard ratio margin of 1.32 (which is equivalent to SF arm having a 1-year LCR of 87%). Hence, this sample size is sufficient to provide evidence of non-inferiority for efficacy.

#### 2^nd^ randomization

The sample size is based on hypothesized QoL differences (mean difference of 5.7) between the intervention arms based on the landmark Basch et al. study, as well as the unpublished standard deviation of 15.6 for EQ-5D-5L scores from our ongoing SABR-COMET-3 study to capture expected standard deviation for our patient population [13, 14]. Group sample sizes of 125 and 125 will provide 80% power to detect a significant difference based on an alpha of 0.05. We will plan an analysis of Arm A and B after 250 patients.

The total sample size for this trial is 598 based on the primary objective of the first randomization.

### Primary endpoints

The primary outcome for the first randomization is HCP-reported toxicity including grade 3-5 adverse events that are possibly, probably, or definitely related to SABR using CTCAE version 5.0. HCP-reported toxicity will be collected at study visits using the AE form. AEs will be compared at the per patient level at any time point post-treatment. Overall rate of AEs is calculated based on the greatest reported severity of SABR-related AEs over the course of the study for each patient. Differences in rates of grade 3 or higher toxicity between groups will be tested using the chi-square test or Fisher’s exact test, as appropriate. Non-inferiority will be assessed using the Farrington-Manning test.

Primary outcome for the 2^nd^ randomization is participant-reported QoL as measured by the EQ-5D-5L. Differences in participant-reported QoL between groups will also be tested using the chi-square test or Fisher’s exact test, as appropriate with non-inferiority assessed using the Farrington-Manning test.

### Survival and secondary endpoints

PFS and OS will be calculated using the Kaplan-Meier method with differences compared using the stratified log-rank test. Pre-planned subgroup analysis will occur based on the stratification factors, and also based on the use of immunotherapy vs. non-immunotherapy systemic agents. A multivariable Cox proportional hazards regression analysis will be used to determine baseline factors predictive of survival endpoints. For the endpoint of time to new metastases, a Fine and Gray competing risk analysis will be used to account for competing risk of death from any cause.

LCR will be calculated using the cumulative incidence function including mortality as competing risk.

### Cost Utility Analysis (CUA)

A CUA will be conducted in line with the Canadian Agency for Drugs and Technologies in Health (CADTH) Guidelines for the Economic Evaluation of Health Technologies [24, 25]. Non-parametric bootstrapping will be used to estimate the 95% confidence intervals (CIs) and to construct a cost-effectiveness acceptability curve. Sensitivity analysis will be conducted by varying the major drivers of costs. All costs will be adjusted to a base year using the healthcare component of the Statistics Canada Consumer Price Index [26] to adjust for price inflation over time. Subsequent incremental cost per unit of OS improvement using OS outcomes will be explored. Although Canada has a single-payer health insurance system, the provincial and territorial governments are responsible for health care administration and delivery. Our analyses will be undertaken from the perspectives of the British Columbia and Ontario provincial Ministries of Health as we expect these provinces to accrue the highest number of patients. We will gain consent from all trial participants to prospectively assess their patient-level records pertaining to the frequency of hospital admissions and the use of targeted- and immuno-therapies. We will use the resource costing method whereby utilization data are collected from existing data sources and then multiplied by unit costs.

### Data Safety Monitoring Committee (DSMC) and interim analyses

There will be four interim analyses. The DSMC will conduct the first interim analysis once 50 patients are accrued. For this analysis, the DSMC will be blinded to the identity of each treatment arm, but OS and toxicity data will be presented for each arm (Arm 1 and 2), and treatment doses and volumes will be presented for SAEs reviewed. There are three planned interim analyses for efficacy in addition to the final analysis. The first two interim analyses are expected to be carried out when the total number of observed study deaths reaches 50 and 100, thus treatment details will be reviewed to determine plausible causation of toxicities. As such, interim efficacy analyses will not be blinded. The DSMC will recommend stopping the trial if there is an OS difference that is statistically significant with a threshold of p < 0.001 using the stratified log-rank test. The third interim analysis will be carried out after 50% accrual, or 250 patients to review QoL for Arm A and B. If the analysis yields significant results, we will select the superior arm and assign all patients moving forward to that group for the remainder of the trial duration. However, if results are not significant, we will continue to randomize patients to either Arm A or B until study completion. This will not affect the 1st randomization between Arms 1 and 2.The final analysis is expected to be carried out 3 years after the enrollment of the last subject.

## Ethical considerations

The Principal Investigator will obtain ethical approval and clinical trial authorization by competent authorities according to local laws and regulations.

### Institutional review board (IRB) / research ethics board (REB)

The protocol (and any amendments), the informed consent form, and any other written information to be given to subjects will be reviewed and approved by a properly constituted Institutional Review Board (IRB)/Research Ethics Board (REB), operating in accordance with the current federal regulations ICH GCP and local regulatory requirements. A letter to the investigator documenting the date of the approval of the protocol and informed consent form will be obtained from the IRB/REB prior to initiating the study. Any institution opening this study will obtain IRB/REB approval prior to local initiation and will be responsible for maintaining approval throughout the duration of the trial. Principal Investigators must provide evidence of IRB/REB approval on an annual basis.

### Informed consent

The written informed consent form is to be provided to potential study patients should be approved by the IRB/ REB and adhere to ICH GCP and the ethical principles that have their origin in the Declaration of Helsinki. The investigator is responsible for obtaining written informed consent from each patient, or if the patient is unable to provide informed consent, the patient’s legally acceptable representative, prior to beginning any study procedures and treatment(s). The investigator should inform the patient, or the patient’s legally acceptable representative, of all aspects of the study, including the potential risks and benefits involved. The patient should be given ample time and opportunity to ask questions prior to deciding about participating in the study and be informed that participation in the study is voluntary and that they are completely free to refuse to enter the study or to withdraw from it at any time, for any reason. The informed consent must be signed and dated by the patient, or the patient’s legally acceptable representative, and by the person who conducted the informed consent discussion. A copy of the signed and dated written informed consent form should be given to the patient or the patient’s legally acceptable representative. The process of obtaining informed consent should be documented in the patient source documents.

### Confidentiality of subject records

The names and personal information of study participants will be held in strict confidence. All study records (case report forms, safety reports, correspondence, etc.) will only identify the subject by initials and the assigned study identification number. The investigator will maintain a confidential subject identification list (Master List) during the course of the study. Access to confidential information (i.e., source documents and patient records) is only permitted for direct subject management and for those involved in monitoring the conduct of the study (i.e., Sponsors and their representatives, representatives of the IRB/REB, and regulatory agencies). The subject’s name will not be used in any public report of the study.

## Data sharing

Protocol data from this study will be pooled with the pending EORTC trial OligoRARE, a randomized trial specifically looking at the impact of SABR in patients with oligometastases from less-common tumour histologies. Anonymized data from patients with non-breast, non-prostate, non-lung and non-colorectal histologies will be shared with EORTC investigators. 

## Protocol amendments and trial publication

Any modifications to the trial protocol must be approved and enacted by the Principal Investigator. Protocol amendments will be communicated to all participating centres, investigators, IRBs, and trial registries by the principal investigator. Any communication or publication of trial results will be led by the principal investigator and is expected to occur within 1 year of the primary analysis. Trial results will remain embargoed until conference presentation of an abstract or until information release is authorized. Authorship of the trial abstract and ultimately the full manuscript will be decided by the principal investigator at the time of submission. Professional writers will not be used for either abstract or manuscript preparation.

## Discussion

The oligometastatic paradigm hypothesizes the existence of an intermediate state between localized and widely disseminated metastatic cancer [1]. Recent randomized data have helped to confirm the existence of the oligometastatic state and demonstrate that ablative therapy - including SABR - improves PFS and OS [27].

By comparing SF and MF SABR in the treatment of oligometastases and oligoprogression, this study aims to determine which SABR regimen is preferrable. If SF SABR is not inferior to MF SABR for the comprehensive treatment of oligometastases, we can offer a recommendation that is less resource intensive for patients and health care systems alike. Additionally, this study will investigate the potential clinical benefit of support provided by a HCP in conjunction with QoL measures administered using electronic entry and will provide further insight into improving patient-reported QoL following SABR.

### Supplementary Information

**Additional file 1: Appendix 1.** BC CANCER PROVINCIAL SABR ORGAN-AT-RISK (OAR) CONSTRAINTS. **Appendix 2.** Reasonable reirradiation SBRT doses to the thecal sac P_max_ following common initial conventional radiotherapy regimens.

## Data Availability

Not applicable.

## Abbreviations

AE: Adverse event
CADTH: Canadian Agency for Drugs and Technologies
CBC: Complete blood count
CBCT: Cone beam computed tomography
CBT: Central Biliary Tract
CEA: Carcinoembryonic antigen
CI: Confidence interval
CNS: Central nervous system
CT: Computed tomography
CTV: Clinical target volume
CTCAE: Common terminology criteria for adverse events
CUA: Cost utility analysis
DSMC: Data safety monitoring committee
ECOG: Eastern cooperative oncology group
EDC: Electronic data capture
ER: Emergency Room
EQ-5D-5L: EuroQoL-5-dimensions-5levels
EQD2: Equivalent Dose in 2 Fractions
FACT-G: Functional assessment of cancer therapy- general
FDG: Fluorodeoxyglucose
GCP: Good clinical practice
GI: Gastro-intestinal
GTV: Gross tumour volume
HRQOL: Health-related quality of life
HCP: Health care provider
ICF: Informed consent form
ICH: International council for harmonization
IRB: Institutional review board
ITV: Internal target volume
LC: Local control
LCR: Lesional control rate
MF: Multiple fraction
MIP: Maximum Intensity Projection (from 4DCT data set)
MinIP: Minimum Intensity Projection (from 4DCT data set)
MRI: Magnetic Resonance Imaging
OAR: Organ at risk
OS: Overall survival
PD: Progressive disease
PET: Positron emitted tomography
PFS: Progression- free survival
PI: Principal Investigator
PSMA: Prostate-specific membrane antigen
POCBP: People of child-bearing potential
PR: Partial response
PRO: Patient reported outcomes
PRV: Planning organ at risk volume
PTV: Planning target volume
QA: Quality assurance
QALY: Quality-adjusted life year
QOL: Quality of life
RCT: Randomized Controlled Trial
REB: Research ethics board
RECIST: Response evaluation criteria in solid tumours
RT: Radiation therapy
SABR: Stereotactic ablative radiotherapy
SAE: Serious adverse event
SF: Single fraction
SOC: Standard of care
SRS: Stereotactic radiosurgery
VMAT: Volumetric modulated arc therapy
