# Supplementary material for: Single vs. multiple fraction non-inferiority trial of stereotactic ablative radiotherapy for the comprehensive treatment of oligo-metastases/progression: SIMPLIFY-SABR-COMET
Source: BMC Cancer. 2024 Feb 3;24:171. doi: 10.1186/s12885-024-11905-7 (PMC10838428; doi:10.1186/s12885-024-11905-7)
Supplement: Supplementary file 1 — Additional file 1: Appendix 1. BC CANCER PROVINCIAL SABR ORGAN-AT-RISK (OAR) CONSTRAINTS. Appendix 2. Reasonable reirradiation SBRT doses to the thecal sac Pmax following common initial conventional radiotherapy regimens. [file 12885_2024_11905_MOESM1_ESM.docx]

## Appendix 1: BC CANCER PROVINCIAL SABR ORGAN-AT-RISK (OAR) CONSTRAINTS

| **OAR** | **SABR ORGAN-AT-RISK (OAR) CONSTRAINTS**  Dmax ≤=0.035cc for all OARs Starred (*) Values are EQD2 equivalent dose conversion from stated reference | | | | | |
| --- | --- | --- | --- | --- | --- | --- |
|  | **Fractions** | | | | | |
|  | **1** | **2** | **3** | **4** | **5** | **8** |

| **Spinal Cord PRV  (2mm on cord)**  **(or Spinal Canal)** | D_max_ ≤ 14 Gy^24,25^ | D_max_ ≤ 19.3 Gy^25^ | D_max_ ≤ 23.1 Gy^25^ | D_max_ ≤ 26.2 Gy^25^ | D_max_ ≤ 28.8 Gy^25^ | D_max_ ≤ 35 Gy^13^ |
| --- | --- | --- | --- | --- | --- | --- |
| ***EQD2/2*** | ***D_max_ ≤ 56 Gy*** | ***D_max_ ≤ 56 Gy*** | ***D_max_ ≤ 56 Gy*** | ***D_max_ ≤ 56 Gy*** | ***D_max_ ≤ 56 Gy*** | ***D_max_ ≤ 56 Gy*** |
| **Spinal Cord PRV /Thecal Sac** (reirradiation) | Refer to: Sahgal A, et al., IJROBP, 82(1):107-116, 2012. DOI: (10.1016/j.ijrobp.2010.08.021) ^11^ | | | | | |
| ***EQD2/2*** | ***Total EQD2/2 ( previous RT + SBRT, no recovery included ) ≤ 70Gy*** | | | | | |
| **Cauda equina (Thecal Sac)** | D_max_ ≤ 16.0 Gy^1^  V14Gy ≤ 5cc Gy^1^ | D_max_ ≤ 20 Gy^1*^  V18.5Gy ≤ 5cc^1*^ | D_max_ ≤ 24 Gy^1^  V21.9Gy ≤ 5 cc^1^ | D_max_ ≤ 29 Gy ^1*^  V27Gy ≤ 5 cc^1*^ | D_max_ ≤ 32 Gy^1^  V30Gy ≤ 5 cc^1^ | D_max_ ≤ 39 Gy^1*^  V36Gy ≤ 5 cc^1*^ |
| ***EQD2/2*** | ***D_max_ ≤ 72 Gy***  ***V56Gy ≤ 5cc*** | ***D_max_ ≤ 60 Gy***  ***V52Gy ≤ 5cc*** | ***D_max_ ≤ 60 Gy***  ***V50Gy ≤ 5cc*** | ***D_max_ ≤ 67 Gy***  ***V60Gy ≤ 5cc*** | ***D_max_ ≤ 67 Gy***  ***V60Gy ≤ 5cc*** | ***D_max_ ≤ 67 Gy***  ***V60Gy ≤ 5cc*** |
| **Sacral plexus** | D_max_ ≤ 16 Gy^1^  V14.4Gy ≤ 5cc^1^ | D_max_ ≤ 21 Gy^1*^ (D_max_ ≤ 26Gy^12^)  V20Gy ≤ 5cc^1*^ | D_max_ ≤ 24 Gy^1^  V22.5 Gy ≤ 5 cc^1^ | D_max_ ≤ 29 Gy ^1*^  V27 Gy ≤ 5 cc^1*^ | D_max_ ≤ 32 Gy^1^  V30 Gy ≤ 5 cc^1^ | D_max_ ≤ 39 Gy^1*^  V36 Gy ≤ 5 cc^1*^ |
| ***EQD2/2*** | ***D_max_ ≤ 72 Gy***  ***V59Gy ≤ 5cc*** | ***D_max_ ≤ 66 Gy***  ***(D_max_ ≤ 97 Gy)***  ***V60Gy ≤ 5cc*** | ***D_max_ ≤ 60 Gy***  ***V53Gy ≤ 5cc*** | ***D_max_ ≤ 67 Gy***  ***V60Gy ≤ 5cc*** | ***D_max_ ≤ 67 Gy***  ***V60Gy ≤ 5cc*** | ***D_max_ ≤ 67 Gy***  ***V60Gy ≤ 5cc*** |
| **Brachial Plexus** | D_max_ ≤ 17.5 Gy^3^  V14Gy ≤ 3cc^3^ | D_max_ ≤ 22 Gy^24*^  V18.5Gy ≤ 3cc^24*^ | D_max_ ≤ 24 Gy ^1^  V20.4Gy ≤ 3cc^1^ | D_max_ ≤ 26 Gy ^4^  V23.6Gy ≤ 3 cc ^3^ | D_max_ ≤ 30.5 Gy ^1^  V27Gy ≤ 3 cc^1^ | D_max_ ≤ 35 Gy ^4^ |
| ***EQD2/2*** | ***D_max_ ≤ 85 Gy***    ***V56Gy ≤ 3cc*** | ***D_max_ ≤ 72 Gy***    ***V52Gy ≤ 3cc*** | ***D_max_ ≤ 60 Gy***  ***V45Gy ≤ 3cc*** | ***D_max_ ≤ 55 Gy***  ***V47Gy ≤ 3cc*** | ***D_max_ ≤ 62 Gy***  ***V50Gy ≤ 3cc*** | ***D_max_ ≤ 56 Gy*** |
| **Heart / Pericardium** | D_max_ ≤ 22 Gy^1^  V16Gy ≤ 15cc^1^ | D_max_ ≤ 25 Gy^1*^  V21Gy ≤ 15cc^1*^ | D_max_ ≤ 30 Gy^1^  V24Gy ≤ 15 cc^1^ | D_max_ ≤ 34 Gy ^3^  V28Gy ≤ 15 cc ^3^ | D_max_ ≤ 38 Gy^1^  V32Gy ≤ 15 cc^1^ | D_max_ ≤ 46 Gy ^2^  V39Gy ≤ 15 cc ^2^ |
| ***EQD2/3*** | ***D_max_ ≤ 110 Gy***  ***V61Gy ≤ 15cc*** | ***D_max_ ≤ 78 Gy***  ***V57Gy ≤ 15cc*** | ***D_max_ ≤ 78 Gy***  ***V53Gy ≤ 15 cc*** | ***D_max_ ≤ 78 Gy***  ***V56Gy ≤ 15 cc*** | ***D_max_ ≤ 81 Gy***  ***V60Gy ≤ 15 cc*** | ***D_max_ ≤ 81 Gy***  ***V61Gy ≤ 15 cc*** |
| **Great Vessels (excl. Pulmonary Artery)** | D_max_ ≤ 37 Gy^3^  V31Gy ≤ 10cc^3^ | D_max_ ≤ 40 Gy^20^ | D_max_ ≤ 44 Gy^20^ | D_max_ ≤ 49.0 Gy^20^ | D_max_ ≤ 51.5 Gy^20^ | D_max_ ≤ 65 Gy |
| ***EQD2/3*** | ***D_max_ ≤ 296 Gy***  ***V210Gy ≤ 10cc*** | ***D_max_ ≤ 184 Gy*** | ***D_max_ ≤ 156 Gy*** | ***D_max_ ≤ 150 Gy*** | ***D_max_ ≤ 137 Gy*** | ***D_max_ ≤ 145 Gy*** |
| **Pulmonary Artery** | D_max_ ≤ 25 Gy^20*^ | D_max_ ≤ 35 Gy^20*^ | D_max_ ≤ 42 Gy^20*^ | D_max_ ≤ 48 Gy^20*^ | D_max_ ≤ 51.5 Gy^20^ | D_max_ ≤ 65 Gy^20*^ |
| ***EQD2/3*** | ***D_max_ ≤ 140 Gy*** | ***D_max_ ≤ 143 Gy*** | ***D_max_ ≤ 143 Gy*** | ***D_max_ ≤ 144 Gy*** | ***D_max_ ≤ 137 Gy*** | ***D_max_ ≤ 145 Gy*** |
| **OAR** | **1** | **2** | **3** | **4** | **5** | **8** |
| **Carotid**  **Artery** | D_max_ ≤ 21 Gy^28*^ | D_max_ ≤ 29 Gy^28*^ | D_max_ ≤ 35 Gy^28*^ | D_max_ ≤ 40 Gy^28*^ | D_max_ ≤ 44 Gy^28^ | D_max_ ≤ 54 Gy^28*^ |
| ***EQD2/3*** | ***D_max_ ≤ 101 Gy*** | ***D_max_ ≤ 102 Gy*** | ***D_max_ ≤ 103 Gy*** | ***D_max_ ≤ 104 Gy*** | ***D_max_ ≤ 104 Gy*** | ***D_max_ ≤ 105 Gy*** |
| **Larynx**  **(extension of trachea)**  **(Priority per MRP)** | D_max_ ≤ 20.2 Gy^1,3**^ | D_max_ ≤ 25 Gy^1*^ | D_max_ ≤ 30 Gy ^1*^ | D_max_ ≤ 34.8 Gy^3^ | D_max_ ≤ 40 Gy^1*^ | D_max_ ≤ 46.3 Gy^1*^ |
| ***EQD2/3*** | ***D_max_ ≤ 94 Gy*** | ***D_max_ ≤ 78 Gy*** | ***D_max_ ≤ 78 Gy*** | ***D_max_ ≤ 81 Gy*** | ***D_max_ ≤ 88 Gy*** | ***D_max_ ≤ 81 Gy*** |
| **PBT and PT  (proximal bronch tree & proximal trachea)** | D_max_ ≤ 20.2 Gy^1,3^ | D_max_ ≤ 25 Gy^1*^ | D_max_ ≤ 30 Gy ^1^ | D_max_ ≤ 34.8 Gy^3^ | D_max_ ≤ 40 Gy^1^ | D_max_ ≤ 46.3 Gy^3*^ |
| ***EQD2/3*** | ***D_max_ ≤ 94 Gy*** | ***D_max_ ≤ 78 Gy*** | ***D_max_ ≤ 78 Gy*** | ***D_max_ ≤ 81 Gy*** | ***D_max_ ≤ 88 Gy*** | ***D_max_ ≤ 81 Gy*** |
| **Pharynx**  **(extension of esophagus)**  **(Priority per MRP)** | D_max_ ≤ 15.4 Gy^1*^ | D_max_ ≤ 21 Gy^1*^ | D_max_ ≤ 27.0 Gy^21*^ | D_max_ ≤ 30 Gy ^3,^ ^21*^ | D_max_ ≤ 35 Gy^1*, 21*^ | D_max_ ≤ 40 Gy ^2*^ |
| ***EQD2/3*** | ***D_max_ ≤ 57 Gy*** | ***D_max_ ≤ 57 Gy*** | ***Dmax ≤ 65 Gy*** | ***Dmax ≤ 63 Gy*** | ***Dmax ≤ 70 Gy*** | ***Dmax ≤ 64 Gy*** |
| **Esophagus** | D_max_ ≤ 15.4 Gy^1^ | D_max_ ≤ 21 Gy^1*^ | D_max_ ≤ 27.0 Gy^21^ | D_max_ ≤ 30 Gy ^3, 21^ | D_max_ ≤ 35 Gy^1, 21^ | D_max_ ≤ 40 Gy ^2^ |
| ***EQD2/3*** | ***D_max_ ≤ 57 Gy*** | ***D_max_ ≤ 57 Gy*** | ***D_max_ ≤ 65 Gy*** | ***D_max_ ≤ 63 Gy*** | ***D_max_ ≤ 70 Gy*** | ***D_max_ ≤ 64 Gy*** |
| **Lungs-GTV** | >1500cc ≤ 7 Gy^1,3^  V11Gy ≤ 10%^8*, 26^  Mean ≤ 4.8 Gy^27*^ | >1500cc≤ 9.5 Gy^1*^  V15 Gy ≤ 10%^3*,8*^  Mean ≤ 6.1 Gy^27*^ | >1500cc ≤ 10.5 Gy^31^  V18 Gy ≤ 10%^3*,8*^  Mean ≤ 7.0 Gy^27*^ | >1500cc≤11.6 Gy^3^  V20 Gy ≤ 10%^3,8*^  Mean ≤ 7.6 Gy^27*^ | >1500cc ≤12.5Gy^1^  V22 Gy ≤ 10%^3*,8*^  Mean ≤ 8.0 Gy^27^ | > 1500c ≤ 14 Gy^3*^  V26Gy ≤ 10%^3*,8*^  Mean ≤ 9.0 Gy^27*^ |
| ***EQD2/3*** | ***>1500cc ≤ 14 Gy***  ***V31Gy ≤ 10%***  ***Mean ≤ 7.5 Gy*** | ***>1500cc ≤ 15 Gy***  ***V32Gy ≤ 10%***  ***Mean ≤ 7.4 Gy*** | ***>1500cc ≤ 14 Gy***  ***V32Gy ≤ 10%***  ***Mean ≤ 7.5 Gy*** | ***>1500cc ≤ 14 Gy***  ***V32Gy ≤ 10%***  ***Mean ≤ 7.4 Gy*** | ***>1500cc ≤ 14 Gy***  ***V33Gy ≤ 10%***  ***Mean < 7.4 Gy*** | ***>1500cc ≤ 13 Gy***  ***V33Gy ≤ 10%***  ***Mean < 7.4 Gy*** |
| **Chest wall and Ribs:** | D_max_ ≤ 26 Gy^7*^  V18Gy ≤ 30cc^6*^ | D_max_ ≤ 36.5 Gy^7*^  V25Gy ≤ 30cc^6*^ | D_max_ ≤ 44 Gy ^7^  V30 Gy ≤ 30 cc^6^ | D_max_ ≤ 50 Gy ^7^  V34 Gy ≤ 30 cc^6^ | D_max_ ≤ 55Gy ^7^  V37 Gy ≤ 30cc^6^ | D_max_ ≤ 68 Gy^7^  V45 Gy ≤ 30 cc^6^ |
| ***EQD2/3*** | ***D_max_ ≤ 151 Gy***  ***V76Gy ≤ 30cc*** | ***D_max_ ≤ 155 Gy***  ***V78Gy ≤ 30cc*** | ***D_max_ ≤ 155 Gy***  ***V78Gy ≤ 30cc*** | ***D_max_ ≤ 155 Gy***  ***V78Gy ≤ 30cc*** | ***D_max_ ≤ 154 Gy***  ***V77Gy ≤ 30cc*** | ***D_max_ ≤ 156 Gy***  ***V78Gy ≤ 30cc*** |
| **Skin** | D_max_ ≤ 21.7Gy^24^  V20.3Gy ≤ 10cc^24^ | D_max_ ≤ 26 Gy^1*^  V24 Gy ≤ 10 cc^1*^ | D_max_ ≤ 33 Gy^1^  V30Gy ≤ 10 cc^1^ | D_max_ ≤ 36 Gy ^3^  V33.2 Gy ≤ 10 cc ^3^ | D_max_ ≤ 39.5 Gy^1^  V36.5 Gy ≤ 10 cc^1^ | D_max_ ≤ 48 Gy^3*^  V44 Gy ≤ 10 cc^3*^ |
| ***EQD2/3*** | ***D_max_ ≤ 107 Gy***  ***V95Gy ≤ 10cc*** | ***D_max_ ≤ 83 Gy***  ***V72 Gy ≤ 10 cc*** | ***D_max_ ≤ 92 Gy***  ***V78 Gy ≤ 10 cc*** | ***D_max_ ≤ 86 Gy***  ***V75 Gy ≤ 10 cc*** | ***D_max_ ≤ 86 Gy***  ***V75 Gy ≤ 10 cc*** | ***D_max_ ≤ 86 Gy***  ***V75 Gy ≤ 10 cc*** |
| **Stomach** | D_max_ ≤ 16.0 Gy^24^  V13Gy ≤ 10cc^24^ | D_max_ ≤ 20 Gy^12^ | D_max_ ≤ 22.2 Gy^1^  (D_max_ ≤25 Gy^14^) | D_max_ ≤ 27^3^ Gy | D_max_ ≤ 32 Gy^1^  (D_max_ ≤ 35 Gy^14^) | D_max_ ≤ 40 Gy ^2^ |
| ***EQD2/3*** | ***D_max_ ≤ 61 Gy***  ***V42Gy ≤ 10cc*** | ***D_max_ ≤ 52 Gy*** | ***D_max_ ≤ 46 Gy***  ***(D_max_ ≤57 Gy)*** | ***D_max_ ≤ 53 Gy*** | ***D_max_ ≤ 60 Gy***  ***(D_max_ ≤70 Gy)*** | ***D_max_ ≤ 64 Gy*** |
| **Duodenum** | D_max_ ≤ 12.4Gy^1^  (D_max_ ≤ 17Gy^24^)  V11.2Gy ≤ 5cc^24^ | D_max_ ≤ 20 Gy^12^ | D_max_ ≤ 22.2 Gy^1^  (D_max_ ≤ 25 Gy^14^) | D_max_ ≤ 29.0 Gy^1*^ | D_max_ ≤ 32 Gy^1,22^  (D_max_ ≤ 35 Gy^14^ ) | D_max_ ≤ 39 Gy ^2^ |
| ***EQD2/3*** | ***D_max_ ≤ 38.2 Gy (D_max_ ≤ 68 Gy)***  ***V32Gy ≤ 5cc*** | ***D_max_ ≤ 52 Gy*** | ***D_max_ ≤ 46 Gy***  ***(D_max_ ≤ 57 Gy)*** | ***D_max_ ≤ 60 Gy*** | ***D_max_ ≤ 60 Gy (D_max_ ≤ 70 Gy*** | ***D_max_ ≤ 61 Gy*** |
| **OAR** | **1** | **2** | **3** | **4** | **5** | **8** |
| **Small Bowel**  **(jejunum / ileum)** | D_max_ ≤ 17 Gy^1*^  V10.2Gy ≤ 5cc^1*^ | D_max_ ≤ 20 Gy^12^ | D_max_ ≤ 25.2 Gy^1, 23^ | D_max_ ≤ 28.5 Gy^23^ | D_max_ ≤ 29.0 Gy^23^  (D_max_ ≤ 35 Gy^1^ ) if PTV close by /  MRP to specify | D_max_ ≤ 40 Gy ^2^ |
| ***EQD2/3*** | ***D_max_ ≤ 68 Gy***  ***V26.9Gy ≤ 5cc*** | ***D_max_ ≤ 52 Gy*** | ***D_max_ ≤ 57 Gy*** | ***D_max_ ≤ 58 Gy*** | ***D_max_ ≤ 51 Gy***  ***(D_max_ ≤ 70 Gy)*** | ***D_max_ ≤ 64 Gy*** |
| **Large Bowel**  **(Colon, Rectum)** | D_max_ ≤ 18.4 Gy^1^ | D_max_ ≤ 20 Gy^12^ | D_max_ ≤ 28.2 Gy ^1^ | D_max_ ≤ 34.5 Gy ^1*^ | D_max_ ≤ 38 Gy ^1^ | D_max_ ≤ 46 Gy^1^ |
| ***EQD2/3*** | D_max_ ***≤ 79 Gy*** | D_max_ ***≤ 52 Gy*** | D_max_ ***≤ 70 Gy*** | D_max_ ***≤ 80 Gy*** | D_max_ ***≤ 81 Gy*** | D_max_ ***≤ 81 Gy*** |
| **Liver (Liver minus GTV)** | > 700 cc ≤ 9.1 Gy^1^ | > 700 cc ≤ 14 Gy^1*^  Mean ≤ 8 - 9 Gy^12^ | >700 cc ≤ 17.4 Gy^31^ | >700 cc ≤ 19 Gy ^1*^ | > 700 cc ≤ 21 Gy^1^ | >700 cc ≤22 Gy^2^ |
| ***EQD2/3*** | ***>700 cc ≤ 22 Gy*** | ***>700 cc ≤ 28 Gy***  ***Mean ≤ 11-14 Gy*** | ***>700 cc ≤ 31 Gy )*** | ***>700 cc ≤ 29 Gy*** | ***>700 cc ≤ 30 Gy*** | ***>700 cc ≤ 25 Gy*** |
| **Central Biliary Tract** | D_max_ ≤ 24 Gy^29*,30*^ | D_max_ ≤ 33 Gy^29*,30*^ | D_max_ ≤ 40 Gy^29*,30*^ | D_max_ ≤ 45 Gy^29*,30*^ | D_max_ ≤ 50 Gy^29,30^ | D_max_ ≤ 61 Gy^29*,30*^ |
| ***EQD2/3*** | ***D_max_ ≤ 130 Gy*** | ***D_max_ ≤ 129 Gy*** | ***D_max_ ≤ 131 Gy*** | ***D_max_ ≤ 128 Gy*** | ***D_max_ ≤ 130 Gy*** | ***D_max_ ≤ 130 Gy*** |
| **Renal Cortex (Kidneys)**  **(R & L combined)** | >200cc ≤ 8.4 Gy^1^ | >200cc ≤12.6 Gy^1*^ | >200cc ≤ 14.4 Gy^31^ | >200cc ≤16.2 Gy^1*^ | >200cc ≤17.5 Gy^1^ | >200cc ≤ 21 Gy^2^ |
| ***EQD2/3*** | ***>200cc ≤ 19 Gy*** | ***>200cc ≤ 23 Gy*** | ***>200cc ≤ 23 Gy*** | ***>200cc ≤ 23 Gy*** | ***>200cc ≤ 23 Gy*** | ***>200cc ≤ 24 Gy*** |
| **Bladder Wall** | D_max_ ≤ 18.4 Gy^1^ | No Available Data. Dose Constraints at RO discretion. | D_max_ ≤ 28.2 Gy^1^ | D_max_ ≤ 34.5 Gy ^1*^ | D_max_ ≤ 38 Gy^1^ | No Available Data. Dose Constraints at RO discretion. |
| ***EQD2/3*** | ***D_max_ ≤ 79 Gy*** | - | ***D_max_ ≤ 70 Gy*** | ***D_max_ ≤ 80 Gy*** | ***D_max_ ≤ 81 Gy*** | - |
| **Penile Bulb** | D_max_ ≤ 34 Gy^1^  V14Gy ≤ 3cc^1^ | No Available Data. Dose Constraints at RO discretion. | D_max_ ≤ 42 Gy ^1^  V21.9 Gy ≤ 3 cc^1^ | No Available Data. Dose Constraints at RO discretion. | D_max_ ≤ 50 Gy^1^  V30 Gy ≤ 3 cc^1^ | No Available Data. Dose Constraints at RO discretion. |
| ***EQD2/3*** | ***D_max_ ≤ 252 Gy***  ***V48Gy ≤ 3cc*** | - | ***D_max_ ≤ 143 Gy***  ***V45Gy ≤ 3 cc*** | - | ***D_max_ ≤ 130 Gy***  ***V54Gy ≤ 3 cc*** | - |
| **Ureter** | D_max_ ≤ 28.5 Gy^24^ | No Available Data. Dose Constraints at RO discretion. | No Available Data. Dose Constraints at RO discretion. | No Available Data. Dose Constraints at RO discretion. | No Available Data. Dose Constraints at RO discretion. | No Available Data. Dose Constraints at RO discretion. |
| ***EQD2/3*** | ***D_max_ ≤ 180 Gy*** | - | - | - | - | - |
| **Femoral Heads (R & L combined)** | V14Gy ≤ 10cc^1^ | No Available Data. Dose Constraints at RO discretion. | V21.9Gy ≤ 10 cc^1^ | V27Gy ≤ 10 cc ^1*^ | V30Gy ≤ 10 cc^1^ | No Available Data. Dose Constraints at RO discretion. |
| ***EQD2/3*** | ***V48Gy ≤ 10 cc*** | - | ***V45Gy ≤ 10 cc*** | ***V53Gy ≤ 10 cc*** | ***V54Gy ≤ 10 cc*** | - |
| **Cochlea** | D_max_ ≤ 12 Gy^18^ | D_max_ ≤ 16.5 Gy^18^ | D_max_ ≤ 20 Gy^18^ | D_max_ ≤ 22.5 Gy^18^ | D_max_ ≤ 25.0 Gy^18^ | No Available Data. Dose Constraints at RO discretion. |
| ***EQD2/3*** | ***D_max_ ≤ 36 Gy*** | ***D_max_ ≤ 37 Gy*** | ***D_max_ ≤ 39 Gy*** | ***D_max_ ≤ 39 Gy*** | ***D_max_ ≤ 39 Gy*** | - |
| **OAR** | **1** | **2** | **3** | **4** | **5** | **8** |
| **Brainstem**  **(not medulla)** | D_max_ ≤ 15 Gy^1^ | No Available Data. Dose Constraints at RO discretion. | D_max_ ≤ 23.1 Gy^1^ | D_max_ ≤ 28 Gy^1*^ | D_max_ ≤ 31 Gy ^1^ | No Available Data. Dose Constraints at RO discretion. |
| ***EQD2/2*** | ***D_max_ ≤ 64 Gy*** | - | ***D_max_ ≤ 56 Gy*** | ***D_max_ ≤ 63 Gy*** | ***D_max_ ≤ 63 Gy*** | - |
| **Optic Pathway** | D_max_ ≤ 12 Gy^17^ | D_max_ ≤ 15.8 Gy^17^ | D_max_ ≤ 19.5 Gy^17^ | D_max_ ≤ 22.5 Gy^17^ | D_max_ ≤ 25 Gy^17^ | No Available Data. Dose Constraints at RO discretion. |
| ***EQD2/2*** | ***D_max_ ≤ 42 Gy*** | ***D_max_ ≤ 39 Gy*** | ***D_max_ ≤ 41 Gy*** | ***D_max_ ≤ 43 Gy*** | ***D_max_ ≤ 44 Gy*** | - |

**Appendix 2: Reasonable reirradiation SBRT doses to the thecal sac P_max_ following common initial conventional radiotherapy regimens**


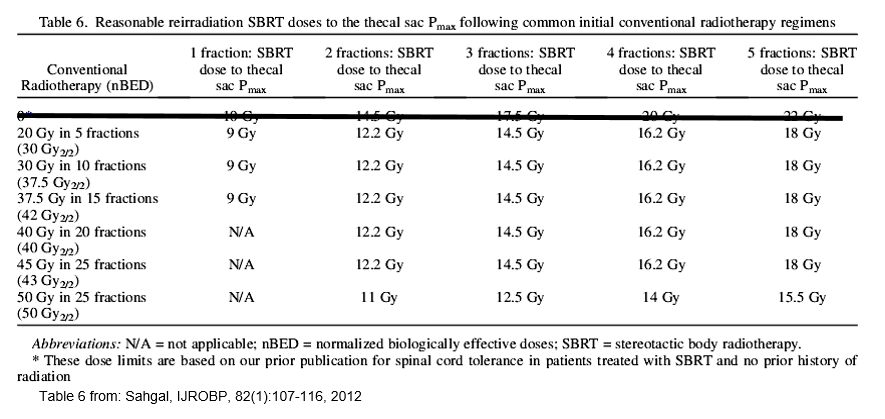


*Table 2 taken from Table 6 of: Sahgal A et al. Reirradiation human spinal cord tolerance for stereotactic body radiotherapy. Int J Radiat Oncol Biol Phys. 2012 Jan 1;82(1):107-16. doi: 10.1016/j.ijrobp.2010.08.021.

1 Benedict SH et al., AAPM TG101, Med.Phys 37(9), 2010 (NB: max pt = 0.035cc). DOI: (10.1118/1.3438081)
^2^ Palma et al., SABR-COMET Trial,, v.1.9, Apr 2015 | The Lancet, 393(10185), 2051-2058 (Appendix). DOI: ( 10.1016/S0140-6736(18)32487-5)
^3^ Videtic et al., RTOG 0915, IJROBP, 93(4) : 757-764, 2015. Supplementary Table E2 DOI: ([10.1016/j.ijrobp.2015.07.2260](https://doi.org/10.1016/j.ijrobp.2015.07.2260))

^4^ Forquer JA et al., Radiother Onc, 93:408-413, 2009. DOI: (10.1016/j.radonc.2009.04.018)

^5^ Karlsson K et al., IJROBP, 87(3): 590-595, 2013. DOI: (10.1016/j.ijrobp.2013.06.2055)

^6^ Dunlap NE et al., IJROBP 76(3): 796-801, 2010. DOI : ( 10.1016/j.ijrobp.2009.02.027)

^7^ Andolino DL et al., IJROBP 80(3): 692-697, 2011. DOI: (10.1016/j.ijrobp.2010.03.020)

^8^ Baker R, IJROBP, 85(1):190-195, 2013. DOI:(10.1016/j.ijrobp.2012.03.041)
^9^ Matsuo Yet al., IJROBP, 83(4): e545-549, 2012. DOI: (10.1016/j.ijrobp.2012.01.018)
^10^ Bongers EM et al., Radiother Onc, 10991): 95-99, 2013. (DOI: [10.1016/j.radonc.2013.10.011](https://doi.org/10.1016/j.radonc.2013.10.011))
^11^ Sahgal A et al., IJROBP, 82(1):107-116, 2012. DOI: (10.1016/j.ijrobp.2010.08.021)
^12^ Sahgal et al.,NCIC CTG Protocol #SC.24. Feb 2, 2017| The Lancet, 22(7), 1023-1033, 2021. .DOI:(10.1016/S1470-2045(21)00196-0), Supplimental

^13^ Sahgal A et al., IJROBP, 85(2):341-7, 2013 ( Table 5; using 5% risk as per Sahgal ; personal communication Feb 2017 )

^14^ Ma, RMK et al, BC Cancer, Provincial Protocol Guidelines for SABR: Hepatocellular Cancer, Version June 27, 2018.

^15^ Velec M, et al, IJROBP, 97(5), 939-946, 2017, DOI: (10.1016/j.ijrobp.2017.01.221)
^16^ Grimm, et al, JACMP, 12(2), 267-292, 2011. DOI: ([10.1120/jacmp.v12i2.3368](https://doi.org/10.1120/jacmp.v12i2.3368))

^17^ Hiniker, et al., Sem Radiat Oncol 26(2), 97-104, 2016. DOI: (10.1016/j.semradonc.2015.11.008)

^18^ Rashid, et al., Sem Radiati Oncol 26(2) 105-111, 2016. DOI: (10.1016/j.semradonc.2015.11.004)

^19^ Kimsey et al., Sem Radiat Oncol 26(2), 129-134, 2016. DOI: (10.1016/j.semradonc.2015.11.003)

^20^ Xue et al, Sem Radiat Oncol 26(2), 135-139, 2016. DOI: (10.1016/j.semradonc.2015.11.001)

^21^ Nuyttens et al., Sem Radiat Oncol 26(2), 120-128, 2016. DOI: (10.1016/j.semradonc.2015.11.006)
^22^ Goldsmith et al., Sem Radiat Oncol 26(2), 149-156, 2016. DOI: (10.1016/j.semradonc.2015.12.002)

^23^ LaCouture et al., Sem Radiat Oncol 26(2), 157-164, 2016. DOI: ([10.1016/j.semradonc.2015.11.009](https://doi.org/10.1016/j.semradonc.2015.11.009))

^24^ Palma et al,SABR-COMET-10, v.2.1,13Oct2021 |BMC Cancer,19,816, 2019. DOI:(10.1186/s12885-019-5977-6)**NOTE :2021 Protocol Update supersedes BMC Cancer**

^25^ Sahgal et al., IJROPB, 110(1), 124-136, 2021. HYTEC - DOI : ([10.1016/j.ijrobp.2019.09.038](https://doi.org/10.1016/j.ijrobp.2019.09.038))

^26^ Liu M et al., BC Cancer Provincial Protocol Guidelines for SABR : Lung, Version May 2021

^27^ Kong et al., IJROBP, 110(1), 172-187, 2021. DOI : (10.1016/j.ijrobp.2018.11.028)

^28^Ling et al., J Radiosurg SBRT, 6, 83-89, 2019. PMID: 31641545 PMCID: PMC6774490

^29^Eriguchi et al.,IJROBP, 85(4). 1006e1011, 2013. DOI: 10.1016/j.ijrobp.2012.09.012

^30^ Diez P, et al. Clinical Oncology 34, 288-300, 2022. DOI: ([10.1016/j.clon.2022.02.010](https://doi.org/10.1016/j.clon.2022.02.010))*Modified

^31^ Moiseenko V, et al. Erratum AAPM TG101, Med Phys, online ahead of print, 2023. DOI: (10.1002/mp.16159)
